# Effects of value and interest intervention on EFL student teachers’ research motivation in the Chinese context

**DOI:** 10.3389/fpsyg.2022.1039473

**Published:** 2022-11-01

**Authors:** Peng Bi, Honggang Liu

**Affiliations:** Department of English, School of Foreign Languages, Soochow University, Suzhou, China

**Keywords:** research motivation, motivation intervention, EFL student teacher, value and interest intervention, mixed methods approach

## Abstract

Language teacher research is conducive to the development of teachers’ teaching skills and professional careers. Thus, many English teacher education programs require student teachers to do research. However, some empirical findings suggest that English as a foreign language (EFL) student teachers lack research motivation. Consequently, finding suitable interventions to increase their research motivation has become increasingly necessary. In light of the importance of research motivation intervention, this study involved designing an experiment to identify the effect of a value and interest intervention including the sharing of positive research experiences to improve student teachers’ research motivation. Quantitative questionnaires and qualitative semi-structured interviews were used to gather evidence on the change in student teachers’ research motivation during the intervention. The interview data revealed that student teachers’ research motivation was influenced by their belief in the value of research to their teaching practice. The experiment results suggested that student teachers’ intrinsic and extrinsic research motivations both increased after the intervention. However, the intervention was not effective in curbing their failure avoidance tendency. Pedagogical implications of the results are discussed at the end of this article.

## Introduction

Language teacher research (be it practitioner research or action research) helps language teachers interpret education policies and understand their teaching contexts and their students’ learning needs (Gilliland, 2018). Teacher research may also develop teachers’ analytical abilities and increase their confidence (Sowa, 2009; Borg, 2010). Put simply, doing research is beneficial for teachers’ pedagogical practice and professional development (Xu, 2013; Hosseini and Bahrami, 2020; Peiser et al., 2022). Thus, the cultivation of research literacy has become a primary concern for both pre-service teachers’ education programs and in-service teachers’ training programs (van Ingen Lauer and Ariew, 2022).

There is a consensus that motivation is among the most important affective-cognitive factors which influence learning, including the acquisition of research skills (Dörnyei and Ushioda, 2010; Yuan et al., 2016; Peiser et al., 2022). Studies on teachers’ research motivation could reveal language teachers’ perceptions of research and explain their engagement in research. There has been a surge in studies investigating the research motivation of language teachers (i.e., Borg and Liu, 2013; Xu, 2013; Yuan et al., 2016; Hosseini and Bahrami, 2020). Those studies reveal that language teachers are not actively engaged in research activities, which may negatively influence their teaching efficacy and professional development. Therefore, it might be advantageous to identify some useful interventions for promoting language teachers’ research motivation. Finding suitable motivation interventions is especially urgent and necessary in China, which has the largest numbers of pre-service and in-service teachers in the world. Moreover, quality improvement is “the theme of teacher education development in China’ (Rao, 2020, p. 95). One useful and effective way to improve teacher quality is to cultivate teachers’ research skills (Bao and Feng, 2022). Thus, the enhancement of student teachers’ research motivation is becoming an essential part of teacher education programs in China. Apart from its practical significance, motivation intervention research can inform motivation and psychological theories (Hulleman and Barron, 2016). Despite the theoretical and practical significance, motivation interventions have received scant attention in the domain of language teaching research.

To address the above-mentioned problem, the current study aimed to identify the effect of a value and interest intervention in enhancing English as a foreign language (EFL) student teachers’ research motivation in China. In other words, this study is based on existing literature and especially the limitations of previous studies. The next section details the relevant studies on language teachers’ research motivation and motivation interventions. Then the research question of this study is presented. What follows is a detailed description of the method of this study, including the participants and context, instruments to measure research motivation and the experiment design. Our results demonstrate increases in student teachers’ intrinsic and extrinsic research motivation after the interventions. However, the value and interest intervention was not effective in helping participants overcome their negative disposition toward doing research. The implications of the results are discussed at the end of this article.

## Literature review

### Language teachers’ research motivation

Research motivation refers to the desire and motive to participate in or withdraw from research activities (Deemer et al., 2010; Yuan et al., 2016; Bahrami and Hosseini, 2022). Several theories have been used to expound on the construct – namely, research motivation, including achievement theory, and self-determination theory (Deemer et al., 2010; Hosseini and Bahrami, 2020). These theories highlight that research motivation is composed of at least three components – intrinsic motivation, extrinsic motivation, and failure avoidance (Deemer et al., 2010; Hosseini and Bahrami, 2020). Intrinsic motivation represents one’s internally driven inclination to do research, such as the aspiration for scientific truth. Extrinsic motivation is the external motive to conduct research, including the desire to gain the respect of colleagues and earn some financial rewards. Failure avoidance means negative feelings about research, focusing on “the reduced research involvement due to fear of failing” in research activities (Hosseini and Bahrami, 2020, p. 4). Different from intrinsic and extrinsic motivations, failure avoidance is the motivation to withdraw from research activities caused by the fear of negative outcomes.

To date, several attempts have been made to explore the status quo of language teachers’ research motivation (e.g., Borg, 2007; Hosseini and Bahrami, 2020). These studies suggest that language teachers have low levels of motivation to do research (be it intrinsic motivation or extrinsic motivation). Particularly, they lack interest in doing research (Xu, 2013) and are unable to recognize the value of academic research and relevance of research to their teaching (Borg, 2009; Medgyes, 2017). The lack of research motivation among language teachers renders it important to find effective interventions to strengthen their motivation. In particular, against the backdrop of the teacher research initiative, the cultivation of research ability, including how to read and interpret published research and how to carry out one’s own studies, has become a required goal for language teacher education programs (van Katwijk et al., 2019; Ndayimirije and Bigawa, 2020). That is, each student teacher is obliged to do research and must complete academic research to graduate at both the bachelor’s and master’s levels. A low level of research motivation would directly influence a student teacher’s research engagement, the quality of their thesis, their research productivity, their well-being and even their future career development (Peng and Gao, 2019; Van Katwijk et al., 2021; Li and Zhang, 2022).

As presented in the Introduction, quality education policies in China require language teachers to be “teachers as researchers” and life-long learners (Rao, 2020). However, studies targeting teachers in China also demonstrate a lack of research motivation and assert the belief that teachers are duty bound to improve their teaching skills rather than research skills (Xu, 2013; Yuan et al., 2016; Bao and Feng, 2022). That is, more attempts should be made to find effective ways to increase student teachers’ research motivation in China. Moreover, due to the large number of teachers in China and the emphasis on teacher quality in national education policies, studies in the Chinese context may have enormous implications for teacher education programs in other countries. The next section will review literature pertaining to motivation intervention.

### Motivation intervention and relevant theoretical approaches

Motivation intervention research is of paramount theoretical and practical importance. Theoretically speaking, the relevant studies can inform us of the components of research motivation and also help us build or modify relevant motivation intervention theories. Practically speaking, these studies can establish a direct link between theoretical motivation constructs and practical pedagogical outcomes (Lazowski and Hulleman, 2016). Moreover, successful interventions which extant studies have identified can be applied in educational practice. Motivation intervention has been embraced for a long time in the domain of educational psychology. Numerous studies have been devoted to seeking effective interventions to enhance students’ learning motivation in education research (for a systematic review, see Lazowski and Hulleman, 2016). Recently, studies have also emerged regarding language learning motivation or academic motivation in general. For example, Alrabai (2016) investigates the extent to which the use of motivational strategies could boost EFL learners’ language learning motivation. The interventions are six motivational strategies targeting “situation-specific learner motivational dispositions,” such as students’ learning autonomy and their perceptions of the usefulness of language learning (Alrabai, 2016, p. 24). Alrabai’s results demonstrate a beneficial effect of the interventions on language learners’ learning motivation and EFL achievement. Similarly, Nawa and Yamagishi (2021) design an experiment to probe the impact of an online gratitude journal intervention on university students’ academic motivation. Their results are also positive, confirming the function of writing gratitude journals in improving students’ academic engagement. To summarize, these studies reveal that successful motivation interventions could not only increase students’ learning motivation but also result in more successful learning outcomes. However, there is a paucity of studies targeting research motivation interventions within the area of language teaching research, not to mention those in the Chinese context. As argued previously, EFL student teachers are not eager to do research, and their low level of research motivation is an urgent matter for teacher educators and policymakers to address in China.

According to Hulleman and Barron (2016), there are two main theoretical approaches to motivation intervention – that is, targeted interventions and comprehensive interventions. The major difference between the two approaches is the motivation types in question. Targeted interventions focus on intervening in one or two components of motivation. Differently, comprehensive interventions treat motivation as a unified construct and are intended to improve participants’ motivation as a whole. Therefore, interventions following the targeted approach are more manageable and less demanding (Hulleman and Barron, 2016). Within a targeted intervention approach, there are four specific types of interventions: expectancy and control beliefs interventions, value and interest interventions, goal interventions and psychological cost interventions. Expectancy and control beliefs interventions are related to students’ attributions of success or failure. Ideally, students should attribute academic success to a more stable factor – namely, the growth of abilities. That is, they should have a growth mindset, which contributes to higher learning motivation. In simple terms, the development of a growth mindset is a major goal of expectancy and control beliefs interventions. Value and interest interventions target students’ perceptions of the value of learning and their interest in learning. This intervention category is intended to lead students to recognize the intrinsic and extrinsic value of learning activities. Goals interventions focus on the use of goal-setting in learning. It is assumed that teachers should lead students to set reasonable goals and encourage them to develop specific behavioral plans. Psychological cost interventions target students’ negative dispositions toward learning. This intervention category focuses more on the alleviation of students’ learning anxiety.

Previous studies contend that language teachers are not keen on doing research mainly because they do not perceive research as a useful and urgent activity for language teachers (Yuan et al., 2016; Medgyes, 2017). In other words, they are not motivated to do research because they are not aware of its value and its relevance to their teaching or professional development. The present study adopted a targeted intervention approach to design an experiment to assess the effectiveness of a value and interest intervention involving role models sharing their research experience. The detailed research question is as follows: To what extent would the value and interest intervention influence EFL student teachers’ research motivation? Both quantitative (i.e., questionnaire survey) and qualitative (i.e., semi-structured interview) data are used to answer this research question. As Creswell (2014) points out, a parallel mixed methods design (i.e., the combined use of quantitative and qualitative data to answer the same research question) would generate more reliable, comprehensive and sound results of the experiment.

## Materials and methods

### Participants and context

This study was conducted in the context of an MA program in English teacher education in a university in eastern China. The main objective of this 2-year program is to foster students’ teaching and research abilities. After graduation, students can be certified to teach English in a secondary school. One requirement of graduation for them is to finish their MA thesis, which should be examined by three experts during the blind review stage. The experiment was carried out in a methodology course of this program in the autumn semester of 2021. Upon successful completion of this course, student teachers can gain a comprehensive understanding of quantitative research methodology and can adopt the suitable method to carry out their own study. To that end, student teachers were also required to read dozens of research papers in this course.

The participants of this study were 45 first-year MA students majoring in English education (44 females and 1 male). Forty students held a bachelor’s degree in English language and literature, while five were non-English majors in their bachelor studies. Although some students had gained research experience during their previous studies, all participants identified as novice researchers. Among those 45 participants, three were selected to participate in a semi-structured interview after the first and last interventions. That is, we used the results of the questionnaire, which participants completed before the experiment, to choose one student teacher with high research motivation (Laura), one with medium research motivation (Eva) and one with low research motivation (Emma) as interview participants to enlarge the representativeness of the qualitative data. The names of the three interviewees used in this article (i.e., Laura, Eva and Emma) are pseudonyms. All the student teachers consented to participate in this study.

### Instruments

Two instruments were used in this study: questionnaire and interview. The questionnaire was the research motivation scale (RMS), developed by Deemer et al. (2010), to measure participants’ pre-test and post-test research motivation (see Supplementary Appendix S1). This scale was originally developed for students majoring in natural science and was later validated by Hosseini and Bahrami (2020) to test language teachers’ research motivation. The RMS was arguably suitable for our participants. As shown in Table 1, it contains 20 items encompassing three motivation components: intrinsic motivation, extrinsic motivation and failure avoidance. The 20 items are scored on a five-point Likert scale (1 = strongly disagree and 5 = strongly agree). One thing to note is that the word *colleague* in some items sounds awkward to Chinese students because they prefer to use *classmate* to refer to each other. Therefore, we changed *colleague* to *classmate* when administering the RMS. This scale had high reliability in the two tests since Cronbach’s alpha of the three motivation categories was above.6 (see Table 1). To minimize the effects of practice, the sequence of items was different in the two tests. As for the two semi-structured interviews, their outlines are listed in Supplementary Appendix S2.

**Table 1:** Overview of the research motivation scale.

Categories	Items	Cronbach’s alpha (pre-test)	Cronbach’s alpha (post-test)
Intrinsic motivation	1; 2; 7; 11; 12; 14; 15; 18; 20	0.810	0.861
Extrinsic motivation	5; 6; 10; 16; 17; 19	0.660	0.854
Failure avoidance	3; 4; 8; 9; 13	0.768	0.844

### Research design

**Figure 1:**
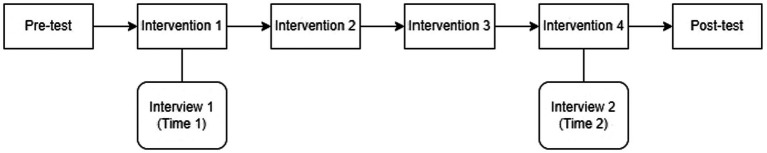
Data collection procedures of the experiment.

This study adopted a pre-experiment design – namely, a pre- and post-test design. Following an interest and value approach, this study used four role models’ sharing of their research experience as the motivation intervention. The role models were selected according to two criteria. First, they needed to have some outstanding research achievements. Three role models have published high-quality papers in refereed journals and one obtained the first prize in a teaching and teacher research competition (see Table 2). All the studies that these four role models have worked on are closely related to language teaching and learning and/or teacher development. Second, to make the role models’ experience sharing compelling for our participants, young researchers who had been doing research for 2–5 years were chosen (see Table 2). This study contained four interventions, whose details are shown in Table 1 and Figure 1. To accentuate the roles of value and interest in the motivation intervention, the four role models were required to emphasize three points as the main contents of experience sharing: (1) their own understanding of research; (2) the reason why they decided to undertake their research and (3) the benefits they have gained from doing research. Each intervention lasted for 30 min including a 10-min-long question-and-answer session. Before the intervention, the details of the experiment were shared with the four role models, including the aim of the experiment and the main contents of the experience sharing.

**Table 2:** Details of the four interventions and four role models.

	Date of intervention	Years of doing research	Representative research outputs
Speaker 1	November 11, 2021	2	One research paper
Speaker 2	November 18, 2021	3	First prize in a teaching and research competition
Speaker 3	November 25, 2021	4	One research paper
Speaker 4	December 2, 2021	5	Four research papers

This study was carried out in three phrases. During the first phase (the pre-test phase), the RMS was administered to participants to gather evidence of their research motivation before the interventions. Participants were not told about the post-test to ensure that they would not deliberately remember their choices in the pre-test. The second phase was the intervention phase. The last phase was the post-test of research motivation which took place immediately after the last intervention. The same questionnaire (i.e., the RMS) was used in the post-test, but the sequence of items differed from that in the pre-test. Forty-five students participated in this study, but only 42 of them finished both the pre- and post-tests. To triangulate the results of the experiment, after the first and last interventions, semi-structured interviews were also conducted with three participants to collect qualitative data on changes in their views about research (see Figure 1). Thematic content analysis was performed on the interview data to ascertain changes in participants’ research motivation (Oattes et al., 2022).

## Results

Table 3 shows the descriptive statistics of participants’ research motivation as measured by the scores of questionnaire items in the pre- and post-tests. On the whole, at the pre-test stage, our participants had a low level of intrinsic research motivation (*M* = 2.937) since they tended to choose *disagree* (score of 2) or *neutral* (score of 3) for corresponding items. The mean score of items from the intrinsic motivation category was 3.732 (see Table 2). On average, the participants either chose *neutral* (score of 3) or *agree* (score of 4) for these intrinsic motivation items. This suggested that they had a mild level of intrinsic motivation. It should be noted that the descriptive statistics of items from the failure avoidance category must be interpreted differently. A higher mean score of these items suggests a higher tendency to withdraw from research activities caused by the fear of failure. Thus, according to Table 3, our participants were likely to avoid negative outcomes when they faced challenges and difficulties in doing research (*M* = 3.533).

**Table 3:** Statistics of three research motivation categories in pre- and post- tests.

	Pre-test		Post-test		*t*(41)	*p*	Cohen’s *d*
	*M*	SD	*M*	*SD*			
Intrinsic	3.732	0.446	3.923	0.398	−3.222	0.002	0.4971
Extrinsic	2.937	0.558	3.156	0.698	−2.058	0.046	0.3176
Failure avoidance	3.533	0.615	3.410	0.620	1.606	0.116	0.2478

The above-mentioned profile of student teachers’ research motivation could be confirmed by data from our first interview.^1^ All three participants agreed that they were pursuing this MA program with the aim of being a teacher rather than a researcher. The following are some illustrative quotes from our interview data: “I chose to attend this MA program in order to get prepared for my teaching career” (Laura); “I am committed to be a teacher much more than a researcher” (Eva); and “the cultivation of teaching skills is the top priority for my MA study” (Emma). At time 1, only Laura was cognizant of the beneficial effects of doing research on her teaching. This fact was consistent with our quantitative results. More specifically, since some students (e.g., Laura) were motivated to do research by their belief that research could improve their teaching, the mean scores of items of the intrinsic motivation category were of a medium level (*M* = 3.732). In contrast, Eva and Emma admitted that research is an “impractical, laborious and theoretical’ thing for them. They were unclear about the necessity of doing research. For example, Emma claimed that “so far, I have not figured out the purpose for us to do research. For me, if it is not compulsory, I am not going to work on it.” Since they believed that doing research is not that necessary and helpful, Eva and Emma even complained that their program’s overemphasis on research literacy would take up time and energy which should be spent on training in teaching skills. They viewed the development of teaching and research skills as contradictory or competitive. Put simply, the three interviewees, and especially Eva and Emma, were not overly enthusiastic about doing research at time 1. Furthermore, the external benefits of doing research, such as earning a scholarship or drawing classmates’ attention, were not appealing to them. Notably, they stated that they were overwhelmed by doing research. For example, Eva commented that “doing research is an extremely difficult thing which is far beyond my current capacity.” Consequently, they tended to “avoid demanding research projects” (see the following quote from Emma).

I will definitely opt for an easy research project. If I find there are many unexpected challenges and difficulties in the project that I have chosen, I will quit… For one thing, I have not mastered a lot of research skills and I am sort of a novice researcher. For another, as a student, I cannot afford the failure of my MA research project.

After the intervention – that is, in the post-test phase, participants’ research motivation presented a quite different profile. Their intrinsic and extrinsic motivations both became higher in that they were more likely to agree with those items from the categories of intrinsic (*M* = 3.923) and extrinsic motivations (*M* = 3.156). Although compared with the pre-test, the mean scores of failure avoidance were lower, participants’ failure avoidance tendency was still quite strong (*M* = 3.410).

**Figure 2:**
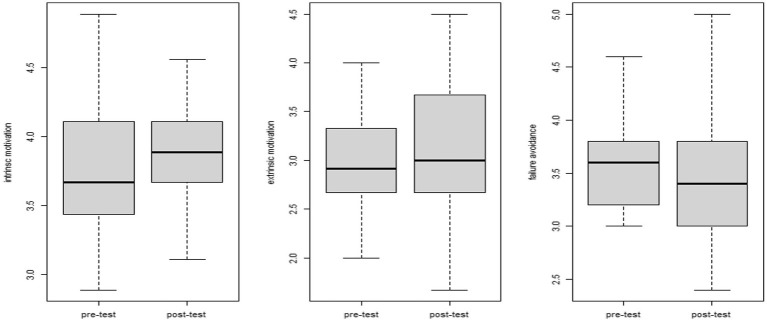
Differences in three research motivation categories between the pre-test and post-test.

Figure 2 shows differences in the three motivation categories between the pre- and post-tests. As Figure 2 clarifies, there were noticeable differences in intrinsic and extrinsic motivations, while failure avoidance tendency was identical on the two tests (see Table 3). Additionally, we utilized a paired samples t-test to check differences in participants’ research motivation between the pre- and post-tests. Paired samples t-tests demonstrated that significant differences in intrinsic [*t*(41) = −3.222; *p* = 0.002; Cohen’s *d* = 0.4971] and extrinsic motivations [*t*(41) = −2.058; *p* = 0.046; Cohen’s *d* = 0.3176] existed between the pre-test and post-test. No significant difference between the two tests was observed for failure avoidance [*t*(41) = 1.607; *p* = 0.116; Cohen’s *d* = 0.2478].

The data of from our second interview align with the quantitative results. After the interventions, all three interviewees formed a new understanding of the value of research. This is exemplified in the views of Eva and Emma: “Now, I come to realize that if the research topics are from our own teaching or learning experience, the research could be closely related to teaching and its results could also be translated into teaching practice easily” (Eva); “From the four speakers” experience sharing, I learned that research can be a down-to-earth and pragmatic thing and provide substantial practical implications for my teaching’ (Emma). Once they recognized the value of research for their teaching, their inner interest in research was largely boosted with the claim that “now I am totally aware of the positive impact of research on my teaching, and I am much more devoted to research in order to solve some practical problems” (Eva). Put simply, the intervention largely boosted participants’ inner interest in research. According to the three interviewees, at time 2, they believed that the external benefits of doing research are rewarding, which may motivate them to engage more in research. However, they confessed that they were still scared of doing research on their own. They were “seized a lot by the fear of failure.” The following quote from Emma may illustrate this point. That is, the interventions did not seem to drastically change their failure avoidance motivation.

Now I know that my research could be valuable and useful only if I choose a topic which is from my own experience or reflection. However, it is literally impossible for me to do so for my MA research project. The reason is simple: I do not have the ability and courage to explore one interesting and meaningful topic regardless of its difficulty. Otherwise, given my insufficient ability, it would probably turn out to be a failure. So, I will opt for a topic from the literature, which may not be so difficult and complex. My point is that compared with the value of the research topic, the difficulty of the research and its probability of failure would be given more attention. Furthermore, for the topic or phenomenon I am interested in, it is not necessary for me to explore it by myself, because I can read relevant studies which could also give me some takeaways for my teaching.

Taken together, the quantitative and qualitative results suggest that a value and interest intervention exemplified by research experience sharing is useful in enhancing EFL student teachers’ research motivation, especially concerning their intrinsic and extrinsic motivations.

## Discussion

Before we interpret the experiment results, one interesting finding from our qualitative data is worth mentioning. That is, student teachers tend to hold the belief that the development of research literacy is a peripheral concern compared with the development of teaching skills in teacher education programs. This finding concurs with previous findings (e.g., van Katwijk et al., 2019; Bao and Feng, 2022). Arguably, it is fair to conclude that student teachers are more teaching-oriented and their attitudes toward research are deeply influenced by their beliefs about the relations between teaching and research. However, although teacher educators and researchers tend to assume that teacher research can empower them to teach in a better and more efficient way, most student teachers do not trust this assumption completely. As our data reveal, some even hold the view that the development of research skills occurs in competition with the development of teaching skills. Along similar lines, van Katwijk et al. (2021, p. 4) remark that “[a]lthough most teacher educators endorse the value of pre-service teacher research, a considerable number of pre-service teachers seem to be skeptical of its relevance for and direct use in the teaching profession.” In light of the above discussion, the ideal interventions to increase student teachers’ research motivation should center on cultivating the thinking that research is beneficial for teaching.

Our experiment results demonstrate the effectiveness of the value and interest intervention in improving student teachers’ research motivation. According to the above discussion, part of the reason to account for the success of the intervention is that the four interventions give participants a full and deep understanding of the value of doing research (Borg, 2010; Yuan et al., 2016; Medgyes, 2017). More specifically, participants came to recognize the value and benefits of doing research for their future teaching from the four role models, leading to an increase in their intrinsic motivation. Likewise, the external benefits obtained by the four role models enabled our participants to comprehend the practical benefits of doing research, such as finding an ideal job and winning a scholarship. Consequently, their extrinsic motivation also exhibited a rising tendency after the four interventions. However, the interventions were not effective for reducing their failure avoidance tendency. One possible explanation is that the value and interest approach we used does not deal with negative dispositions of research motivation. As Hulleman and Barron (2016) suggest, the psychological cost intervention approach seems more suitable to tackle failure avoidance. In this respect, our results lend empirical support to Hulleman and Barron (2016) motivation intervention theory. That is, the targeted intervention approach is only effective for some components of motivation depending on the specific approach being used. In this case, the value and interest approach was primarily helpful in enhancing student teachers’ intrinsic and extrinsic research motivations. This study further demonstrates the importance of experimental work in building motivation theory. Hulleman and Barron (2016, p. 182) also assert that “additional experimental tests of theory offer a more rigorous test of the theory, moving beyond the information that can be learned from interviews, observations and correlational studies.” In a similar vein, more experimental work is warranted in the domain of language teachers’ research motivation.

## Conclusion

This study attempted to clarify the effects of a value and interest intervention in enhancing EFL student teachers’ research motivation. Our analysis of the quantitative and qualitative data yielded two major findings. First, student teachers’ research motivation tends to be greatly influenced by their beliefs about the value of research for their teaching. If they assume that research is conducive to their teaching, they will have a greater inner interest in research (i.e., higher intrinsic motivation) and vice versa. Second, an intervention instantiated by research experience sharing is effective in improving student teachers’ intrinsic and extrinsic research motivations.

The results of this study have significant implications for teacher educators and education program administrators. Firstly, in terms of intrinsic motivation, teacher educators must determine ways to raise students’ awareness of the importance of research and the relevance of research to their future teaching career. For example, apart from experience sharing, leading students to read relevant literature on pedagogical implications may also enable students to realize the function of language teacher research, thereby increasing their intrinsic motivation. Meanwhile, professors could employ some output-oriented teaching methodologies, such as project-based methods, to help students realize the importance of research for their future teaching career and arouse their interest in carrying out research. The goal of enhancing student teachers’ intrinsic motivation is to make them resilient (Chu and Liu, 2022; Liu and Chu, 2022) in their future teaching career. Secondly, regarding extrinsic motivation, administrators should make full use of these external factors to motivate student teachers to do research. For instance, a more vigorous and viable assessment system which incorporates research achievements should be designed to encourage student teachers to devote more to research. They can also design some relevant policies and implement some useful initiatives to build a more thriving and vibrant learning and research community among students. To that end, peer learning may enhance student teachers’ motivation to do research.

As mentioned previously, this study further illustrates the importance of experimental work in building and testing motivation theory. However, it should be noted that there was a minor limitation regarding the experiment design of this study. Since the number of students is limited within English education programs in virtually all Chinese universities, it is impractical to divide participants into two groups (experiment and control groups). That means the pre-experiment design had to be used in this study. Compared with the quasi-experiment design, the pre-experiment design may give rise to some erroneous variables, such as the influence of supervisors on participants’ research motivation. Such erroneous variables, however, may not have influenced our results substantially because research motivation is quite a stable cognitive-affective construct. Additionally, the sample size of this experiment was quite small. Future studies may include more participants to enlarge the representativeness of the experiment results.

## Data availability statement

The original contributions presented in the study are included in the article/Supplementary material, further inquiries can be directed to the corresponding author.

## Ethics statement

The studies involving human participants were reviewed and approved by School of Foreign Languages, Soochow University. The patients/participants provided their written informed consent to participate in this study.

## Author contributions

PB: conceptualization, data collection and analysis, writing, revision, and funding. LH: conceptualization, data analysis, revision, and funding. All authors contributed to the article and approved the submitted version.

## Funding

This study was supported by Jiangsu Shuangchuang Talent Program 2021 (Grant No. JSSCBS20210636), Fundamental Research Program, Soochow University (Grant No. 21XM1007), and Project of Developmental Features of the Language Ability by Chinese Multilingual Second Language Learners-Youth Team Funding of Northeast Normal University 2021 (Grant number: 2021QT004).

## Conflict of interest

The authors declare that the research was conducted in the absence of any commercial or financial relationships that could be construed as a potential conflict of interest.

## Publisher’s note

All claims expressed in this article are solely those of the authors and do not necessarily represent those of their affiliated organizations, or those of the publisher, the editors and the reviewers. Any product that may be evaluated in this article, or claim that may be made by its manufacturer, is not guaranteed or endorsed by the publisher.

## Supplementary material

The Supplementary material for this article can be found online at: https://www.frontiersin.org/articles/10.3389/fpsyg.2022.1039473/full#supplementary-material

## Footnotes

^1^It should be noted that the first interview took place after the first intervention because we aimed to capture the change in participants’ research motivation. As for retrospective questions related to students’ original research motivation, the interviewers made it clear that interviewees should describe their true thoughts on research before the first intervention.
