# Supplementary material for: Effects of value and interest intervention on EFL student teachers’ research motivation in the Chinese context
Source: Front Psychol. 2022 Nov 1;13:1039473. doi: 10.3389/fpsyg.2022.1039473 (PMC9663849; doi:10.3389/fpsyg.2022.1039473)
Supplement: Supplementary file 1 [file Data_Sheet_1.docx]

**Supplementary materials**

Supplementary Appendix S1

Research Motivation Scale

1. I have a general feeling of well-being when I’m involved in research.

2. I conduct research for the joy of it.

3. I want to be recognized by my classmates as a competent researcher.

4. I want to be recognized by my classmates for conducting sound research.

5. I want to pursue less difficult research projects that I know will guarantee a successful outcome.

6. When the preliminary results of my research have not met my expectations, I want to cut my losses and move on to the next project.

7. Conducting research provides me with feelings of satisfaction.

8. I conduct research to earn the respect of my classmates.

9. I want to leave my mark on my field.

10. I sometimes want to avoid difficult research projects because I’m concerned that I may fail.

11. I love to learn new things through research.

12. I have a need to understand scientific phenomena.

13. I want to receive awards for my scientific accomplishments.

14. I feel great pleasure when I’ve learned something new in my area of research.

15. Research in and of itself is enjoyable to me.

16. I sometimes want to give up when my research is not proceeding as I would like.

17. I want to focus more of my energy on other research projects when the current project I am working on is not processing as expected.

18. I enjoy doing research for its own sake.

19. I want to avoid pursuing difficult research projects that might result in a negative outcome (e.g., lack of significant findings, not accepted for publication, etc.).

20. Time seems to fly by when I’m conducting research.

Supplementary Appendix S2

Outline of interview 1

1. Why do you choose to purse your MA study?

2. Please describe your learning and research experience in this MA programme.

3. What is your future career plan and why?

4. What is your understanding towards research before the experiment starts?

5. To what extent will you engage in research activities and what are some reasons?

6. What is your view on the relation between teaching and research before the experiment starts?

7. What is your takeaway from today’s experience sharing?

Outline of interview 2

1. What have you learned from the four models?

2. Could you share with us some impressive things related to the four experience sharing sessions?

3. What is your understanding towards research now? Any changes compared with your previous understanding and why?

4. What is your view on the relation between teaching and research? Any changes compared with your previous understanding and why?

5. What are the possible reasons for you to do or not to do research?

6. Have you experienced some difficulties in your M.A. study?

7. Could you share with us some of your research experience?
